# Reactive Compatibilization of Polyglycolic Acid/Polyurethane Blends for an Improved Strength–Toughness Balance

**DOI:** 10.3390/polym18202471

**Published:** 2026-10-10

**Authors:** Xiuying Zhao, Zhi Chen, Xin Wang, Qingsheng Dong, Lin Liu, Jizhu Liang, Dexian Yin

**Affiliations:** 1 State Key Laboratory of Organic-Inorganic Composites, Beijing University of Chemical Technology, Beijing 100029, China; 2 Jiangyin Haida Rubber and Plastic Co., Ltd., Wuxi 214423, China

**Keywords:** polyglycolic acid, thermoplastic polyurethane, reactive compatibilization, strength–toughness balance

## Abstract

Polyglycolic acid (PGA) possesses superior mechanical strength but suffers from inherent brittleness, which severely limits its application scope. In this work, we synthesized a high-toughness poly(ε-caprolactone)-based thermoplastic polyurethane (TPU) to toughen PGA, employing a multifunctional epoxy chain extender (ADR) as a reactive compatibilizer. Melt blending PGA with TPU successfully induced a brittle-to-ductile transition, evolving the phase morphology from a sea-island structure to a co-continuous architecture. While the binary PGA/TPU blend achieved a remarkable notched impact strength of 21.7 kJ m^−2^ (approximately 7.5 times that of neat PGA), the weak interfacial adhesion resulted in a drastic sacrifice of tensile strength, which plummeted from 131.9 MPa to 37.1 MPa. To overcome this trade-off, ADR was introduced to promote chain extension, branching, and enhanced interfacial interactions during melt blending. Consequently, the representative blend achieved an improved strength–toughness balance, exhibiting a tensile strength of 75.1 MPa and a robust notched impact strength of 16.9 kJ m^−2^. The combination of high mechanical strength and enhanced impact resistance endows the developed PGA-based blends with considerable potential for advanced load-bearing and impact-resistant polymer components. In particular, these characteristics may enable their use in emerging applications such as compliant structural components, protective elements, and mechanically robust parts for biomimetic robotic systems.

## 1. Introduction

The escalating environmental crisis driven by the accumulation of petroleum-derived plastics has accelerated the search for sustainable and biodegradable alternatives [1,2,3]. Polyglycolic acid (PGA) has emerged as a promising biodegradable polyester due to its relatively rapid degradation into non-toxic products under appropriate physiological and environmental conditions. Structurally, the tight chain packing of its simple two-carbon repeat unit endows PGA with high crystallinity (30–50%) and superior mechanical strength [4,5,6]. Furthermore, PGA exhibits a degradation rate comparable to cellulose, fully decomposing within 3–4 months under industrial composting conditions [7,8,9]. However, its inherent brittleness, characterized by a low elongation at break, severely restricts its broader industrial applicability [10,11,12]. Consequently, developing effective toughening strategies is critical to unlocking the full market potential of PGA.

Various strategies have been developed to overcome the intrinsic brittleness of PGA, including copolymerization, plasticization, blending with flexible polymers, and reactive compatibilization. Among these approaches, melt blending with flexible polymers or elastomers is particularly attractive because of its simple processing procedure and high industrial scalability [13]. Previous studies have therefore incorporated flexible biodegradable polymers, including poly(ε-caprolactone) and poly(butylene adipate-co-terephthalate), into PGA to improve its ductility and impact resistance [14,15]. However, these flexible polyester modifiers mainly rely on the introduction of ductile phases to promote plastic deformation, and the resulting improvement in toughness is often accompanied by a reduction in tensile strength due to limited load-transfer capability and weakened matrix continuity. Therefore, developing a toughening phase that can simultaneously provide efficient energy dissipation and maintain structural reinforcement remains a critical challenge for PGA modification.

Thermoplastic polyurethane (TPU) elastomers provide a distinctive alternative because of their segmented molecular architecture consisting of flexible soft segments and rigid hard segments. The soft segments enable large-scale deformation and energy dissipation, while the hard segments provide physical reinforcement and contribute to mechanical strength. Compared with conventional flexible polyester modifiers such as PCL and PBAT, TPU therefore offers potential advantages in constructing a more balanced strength–toughness profile. Nevertheless, the limited compatibility between PGA and TPU, arising from differences in molecular structure and intermolecular interactions, may still lead to phase separation and insufficient interfacial stress transfer. Therefore, regulating the interfacial structure of PGA/TPU blends is essential for achieving enhanced mechanical performance.

Reactive compatibilization using multifunctional epoxy-based chain extenders provides an effective route to regulate interfacial interactions in immiscible polyester blends. During melt processing, the epoxy groups of ADR may undergo ring-opening reactions with terminal functional groups of polyester chains, regulating molecular architecture and interfacial interactions through possible chain extension and branching reactions. These structural changes can increase molecular architecture complexity, suppress coalescence of the dispersed phase, refine phase morphology, and strengthen interfacial interactions. Consequently, epoxy-functional reactive compatibilizers have been widely employed to improve the compatibility and mechanical performance of immiscible polymer blends. Furthermore, recent studies on sustainable thermoplastic systems have demonstrated that mechanical performance should be interpreted together with thermal behavior and phase morphology, since interfacial interactions, phase dispersion, and crystallization behavior can collectively govern the macroscopic properties of polymer blends and composites [16].

In this work, we propose a reactive compatibilization strategy to achieve an improved strength–toughness balance in PGA-based blends. Specifically, a poly(ε-caprolactone)-based TPU was synthesized as the toughening phase, utilizing its segmented structure to provide both deformation-induced energy dissipation and structural reinforcement. To further regulate the PGA/TPU interface, a multifunctional epoxy chain extender (Joncryl ADR 4468, designated as ADR) was incorporated during melt blending to promote chain extension, branching, and enhanced interfacial interactions. We systematically investigated the effects of TPU loading on the toughening behavior and ADR dosage on the phase morphology, thermal behavior, and mechanical performance of the blends. Unlike conventional PGA/PCL and PGA/PBAT blends that mainly rely on flexible-phase toughening, the present PGA/TPU/ADR system combines the unique segmented structure of TPU with reactive interfacial regulation, enabling improved impact resistance and tensile toughness of the selected PGA/TPU formulation. This work provides a strategy for designing high-performance polyester blends with balanced mechanical properties.

## 2. Experimental

### 2.1. Materials

PGA was supplied by Inner Mongolia Pujing Polymer Material Technology Co., Ltd. (Inner Mongolia, China), and poly(ε-caprolactone) diol was obtained from Masaru Chemical Co., Ltd. (Tokyo, Japan). The 1,4-butanediol (BDO) and 1,6-hexamethylene diisocyanate (HDI) were purchased from Shanghai Macklin Biochemical Technology Co., Ltd. (Shanghai, China) with a purity of ≥99%. The ADR was purchased from BASF (Ludwigshafen, Germany); it has a number-average molecular weight (M_n_) of approximately 6800 g mol^−1^ and an epoxy equivalent weight of roughly 310 g mol^−1^. Prior to melt blending, PGA was dried in a vacuum oven at 80 °C for 12 h, while the synthesized TPU was vacuum-dried at 60 °C for 12 h to minimize the influence of absorbed moisture during high-temperature processing.

### 2.2. Synthesis of the TPU

A poly(ε-caprolactone) diol with a molecular weight of 1250 g mol^−1^ was used as the soft segment. The designed hard-segment content of the TPU was 45%. The molar ratio of poly(ε-caprolactone):HDI:BDO was 1:4.31:3.31, corresponding to an overall NCO/OH equivalent ratio of 1.00. No catalyst was used during either the prepolymerization or chain-extension stages. Poly(ε-caprolactone) was first dried under vacuum (−0.1 MPa) at 120 °C for 1.5 h and subsequently cooled to 65 °C. The system was then purged with nitrogen and maintained under a nitrogen atmosphere. HDI was added to initiate the prepolymerization reaction at 75 °C for 1.5 h. Subsequently, BDO was introduced to complete the chain-extension reaction at the same temperature for 0.5 h. The synthesized TPU was then cured at 90 °C for 12 h to promote completion of the residual reactions. The detailed formulation is provided in Table S1, and the synthetic route is illustrated in Figure S1. The chemical structure and mechanical response of the synthesized TPU are presented in Figure S2 and Figure S3, respectively.

### 2.3. Fabrication of PGA/TPU and PGA/TPU/ADR Blends

Melt blending was performed using a Haake Rheomix internal mixer (Thermo Fisher Scientific, Karlsruhe, Germany) at 220 °C with a rotor speed of 100 rpm. PGA was charged into the mixer first, followed by the addition of TPU once the temperature stabilized at 220 °C. For the binary blends, the mixture was blended for 3 min before being discharged. These blends, with PGA/TPU mass ratios of 100/0, 90/10, 80/20, 70/30, 60/40, and 50/50, are designated as PGA/TPU0, PGA/TPU1, PGA/TPU2, PGA/TPU3, PGA/TPU4, and PGA/TPU5, respectively (Table S2).

For the ternary blends incorporating the reactive compatibilizer, ADR was introduced after the initial melting of PGA and TPU, and the system was mixed for an additional 4 min to facilitate the interfacial reaction. These PGA/TPU/ADR blends were prepared using a fixed PGA/TPU mass ratio of 80/20. ADR was added at 0.3, 0.6, 0.9, 1.2, or 1.5 parts per hundred parts (phr) by mass of the PGA/TPU matrix, as detailed in Table S3. Finally, all compounded samples were injection-molded into standard test specimens using a WZS10D micro-plunger injection molding machine (Shanghai Xinshuo Precision Machinery Co., Ltd., Shanghai, China) with a barrel temperature of 230 °C, a mold temperature of 70 °C, a hydraulic oil pressure of 0.5 MPa, and a holding time of 8 s.

### 2.4. Characterizations

#### 2.4.1. Chemical Structure Analysis

An FTIR spectrum was acquired using a Tensor II spectrometer (Bruker, Ettlingen, Germany). The measurements were conducted in attenuated total reflectance mode across a wavenumber range of 3500 to 600 cm^−1^.

#### 2.4.2. Rheological and Processing Behavior

The torque–time evolution of the PGA/TPU and PGA/TPU/ADR systems was monitored using a Haake mixer (Thermo Fisher Scientific, Karlsruhe, Germany) to evaluate the melt processing behavior and interfacial reactions. These tests were performed at a temperature of 220 °C and a rotor speed of 100 rpm. Additionally, the melt flow rate (MFR) of the blends was determined using a melt flow indexer at 230 °C under a standard load of 2.16 kg.

#### 2.4.3. Thermal Properties

Differential scanning calorimetry (DSC) was employed to analyze the crystallization and melting behaviors of the blends under a nitrogen atmosphere. To eliminate previous thermal history, the samples were first held at 250 °C for 3 min. Subsequently, the samples were cooled to 0 °C, held for 3 min, and then reheated to 250 °C. Both the heating and cooling rates were maintained at 10 °C min^−1^. The degree of crystallinity (*χ_c_*) of the PGA phase was calculated using the following equation:

$$\chi_{c}=\;\frac{\Delta H_{m}}{\omega_{\mathrm{PGA}}\times \Delta H_{m}^{0}}\times 100\%$$

where Δ*H_m_* represents the melting enthalpy derived from the second heating scan, ω_PGA_ denotes the weight fraction of PGA in the binary or ternary blends, and Δ*H_m_*^0^ is the theoretical melting enthalpy of 100% crystalline PGA, taken as 198.2 J g^−1^ [17,18].

The thermal stability of the blends was evaluated via thermogravimetric analysis (TGA) using a TGA2 instrument (Mettler Toledo, Greifensee, Switzerland). The measurements were conducted across a temperature range of 30 to 800 °C at a heating rate of 10 °C min^−1^ under a nitrogen atmosphere.

#### 2.4.4. Morphological Analysis

To investigate the phase morphology, the samples were cryo-fractured in liquid nitrogen. The exposed surfaces were subsequently treated with N,N-dimethylformamide (DMF) for 4 days to visualize the dispersed TPU-rich phase based on the different solvent affinities of TPU and PGA toward DMF. Prior to imaging, the etched surfaces were sputter-coated with gold, and the morphological features were examined using scanning electron microscopy (SEM) at an accelerating voltage of 5 kV.

#### 2.4.5. Mechanical Testing

Tensile properties were evaluated using injection-molded Type IV specimens in accordance with the ASTM D638-22 [19] standard, with a crosshead speed of 20 mm min^−1^ on a SANS universal testing machine. Notched Izod impact performance was determined according to the GB/T 1843-2008 [20] standard using injection-molded Type A specimens (80 × 10 × 4 mm^3^) on a cantilever-beam pendulum impact tester equipped with a 2.75 J pendulum. To ensure data reliability, at least five replicates were tested for each sample, and the mean values were reported.

## 3. Results and Discussion

### 3.1. Effect of TPU Content on the Toughening of PGA/TPU Blends

#### 3.1.1. Processability of PGA/TPU Blends

**Figure 1:**
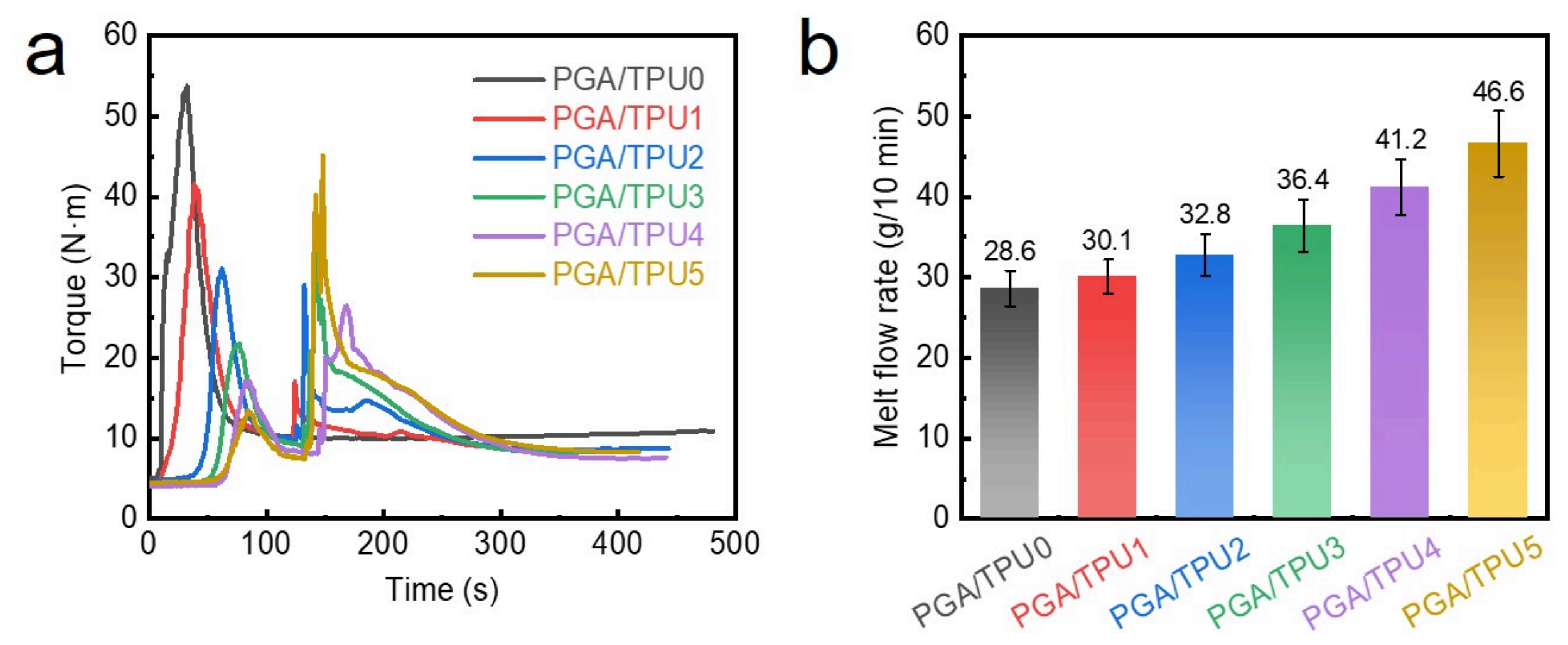
(**a**) Torque evolution versus time of the PGA/TPU blends; (**b**) MFR of the PGA/TPU blends.

The melt processing characteristics of the blends were evaluated via MFR measurements. Neat PGA exhibited an MFR of 28.6 g/10 min. Upon the incorporation of TPU, a distinct dilution effect was observed in the melt, which effectively reduced the viscosity of the system. As illustrated in Figure 1, increasing the TPU fraction amplified this effect, leading to a monotonic increase in MFR. Consequently, the MFR reached 46.6 g/10 min for the PGA/TPU5 blend. This trend confirms that the flexible TPU phase enhances the flowability of the rigid PGA matrix during processing.

#### 3.1.2. Thermal Properties of PGA/TPU Blends

**Figure 2:**
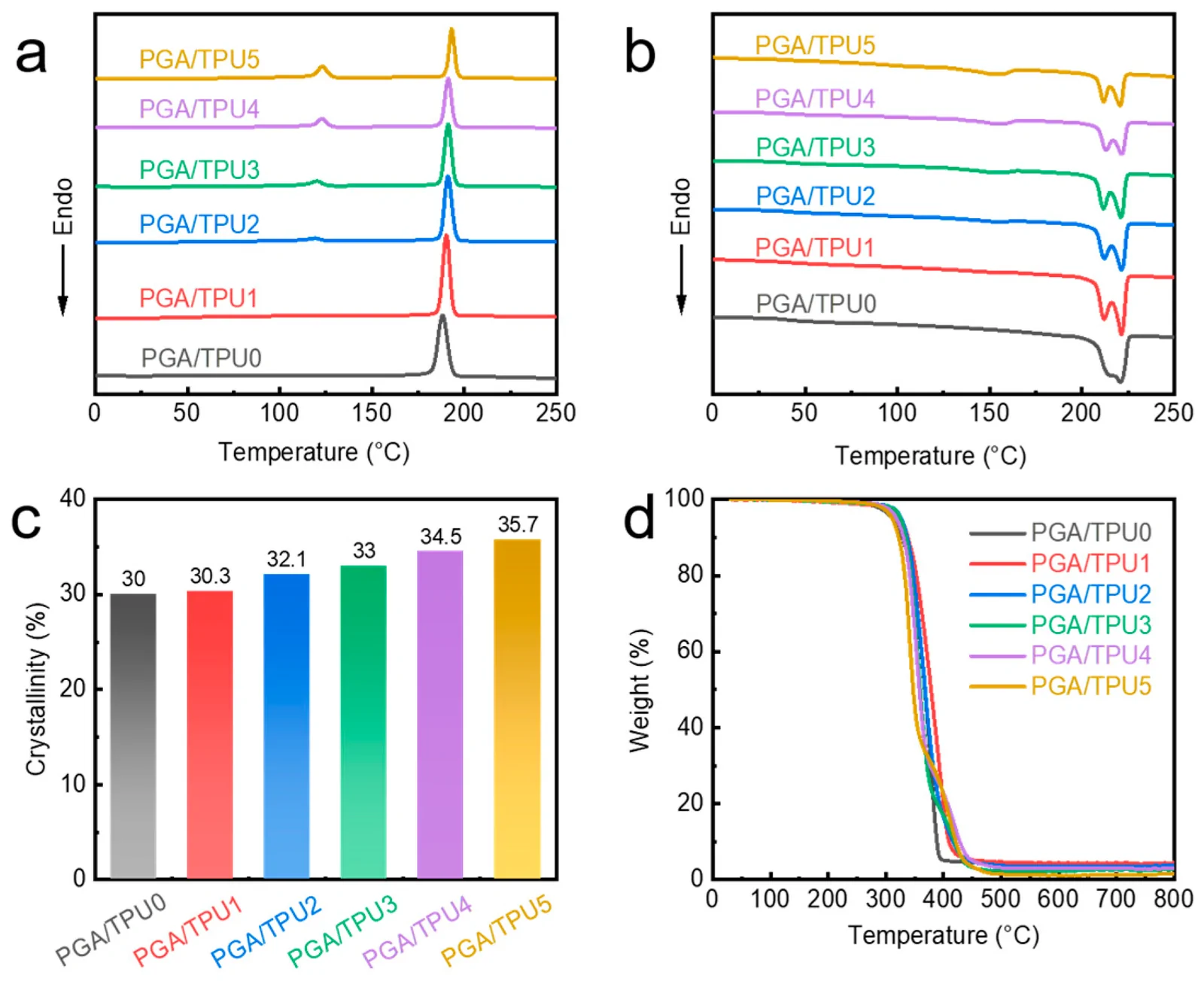
(**a**) Cooling DSC curves of PGA/TPU blends; (**b**) Second heating DSC curves; (**c**) Crystallinity variations; (**d**) TGA thermograms.

The crystallization behavior of the PGA matrix is influenced by several factors, including chain mobility, phase morphology, interfacial interactions, and crystal growth processes. As shown in Figure 2a, the incorporation of TPU shifts the crystallization temperature (*T_c_*) of the PGA phase to higher values. This shift indicates a modification of the crystallization behavior of PGA after TPU incorporation, which may arise from the combined effects of altered chain mobility, phase morphology, and interfacial interactions. Figure 2b displays the second-heating DSC curves, in which two distinct melting endotherms are observed. The higher-temperature endotherm is assigned to the melting of the PGA crystalline phase, whereas the lower-temperature endotherm is mainly associated with the melting of crystalline PCL-based soft-segment domains within the TPU phase. Neat PGA exhibits a melting temperature (*T_m_*) of 220.8 °C, as shown in Table S4. The persistence of these separate thermal transitions indicates that the PGA-rich and TPU-related phases retain largely distinct thermal behaviors after melt blending. With increasing TPU content, the Δ*H_m_* of PGA/TPU blends decreases monotonically due to the dilution effect of the amorphous TPU phase. Specifically, Δ*H_m_* drops from 59.5 J g^−1^ for neat PGA to 35.4 J g^−1^ for the blend containing 50 wt% TPU. Interestingly, despite the dilution effect, the *χ_c_* of the PGA matrix shows a gradual upward trend with increasing TPU loading (Figure 2c and Table S4). The gradual increase in *χ_c_* with increasing TPU content suggests that TPU incorporation modifies the crystallization environment of the PGA matrix. This behavior may be associated with changes in chain mobility and phase morphology, while the PGA/TPU interfaces may also provide sites that facilitate crystallization. However, the present DSC results alone cannot distinguish the relative contributions of these effects.

Figure 2d and Table S4 illustrate the thermal stability of the blends evaluated via TGA. The mass-loss profiles exhibit a characteristic two-stage degradation behavior. The initial stage corresponds to the decomposition of the PGA matrix [21], while the second stage is attributed to the thermal breakdown of the TPU phase [22]. While the dispersed TPU promotes PGA crystallization and theoretically enhances thermal resistance, the overall thermal stability of the blends declines with increasing TPU loading. Nevertheless, all blends exhibited 5% weight loss temperature (*T_5%_*) values above 320 °C, which is considerably higher than the processing temperatures used for melt blending (220 °C) and injection molding (230 °C). The sufficient temperature margin suggests that the prepared blends possess adequate thermal stability under the applied processing conditions.

#### 3.1.3. Phase Morphology and Fracture Mechanism

**Figure 3:**
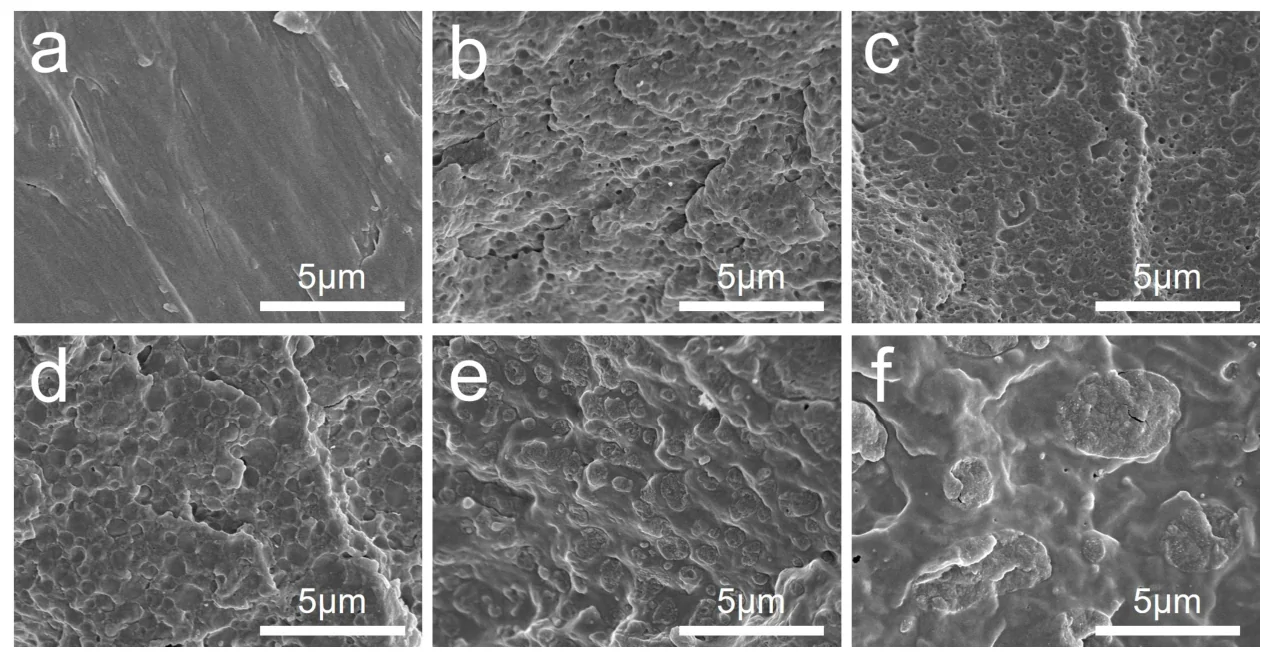
SEM micrographs of cryo-fractured surfaces of PGA/TPU blends after DMF etching: (**a**) PGA/TPU0; (**b**) PGA/TPU1; (**c**) PGA/TPU2; (**d**) PGA/TPU3; (**e**) PGA/TPU4; (**f**) PGA/TPU5.

To visualize the phase distribution and interfacial structure, the cryo-fractured surfaces of the blends were treated with DMF. The resulting etched morphology provides qualitative information regarding the distribution of TPU-rich domains. Figure 3 presents the SEM micrographs of the etched surfaces. Neat PGA exhibits a smooth and featureless fracture surface (Figure 3a), characteristic of typical brittle failure. Upon the incorporation of TPU (10–30 wt%), the etched surfaces reveal numerous spherical cavities, which are associated with the removal of TPU-rich domains during DMF treatment, indicating the dispersed-phase morphology within the continuous PGA matrix (Figure 3b–d). With increasing TPU content, a distinct enlargement of these dispersed domains is observed. This trend signifies phase coarsening driven by the thermodynamic incompatibility between the rigid polyester matrix and the flexible elastomer.

When the TPU loading reaches 40 wt% and 50 wt% (Figure 3e,f), the morphology undergoes a transition from a typical sea-island structure to a co-continuous phase architecture. The isolated domains begin to coalesce, forming an interconnected network. This morphological evolution plays a pivotal role in the toughening mechanism [23]. The dispersed TPU particles act as stress concentrators that induce cavitation and massive shear yielding in the matrix upon impact. Consequently, the fracture surfaces of the blends become significantly rougher compared to neat PGA, exhibiting pronounced plastic deformation and stress-whitening, which are hallmarks of enhanced energy dissipation and improved impact resistance.

#### 3.1.4. Mechanical Properties of PGA/TPU Blends

**Figure 4:**
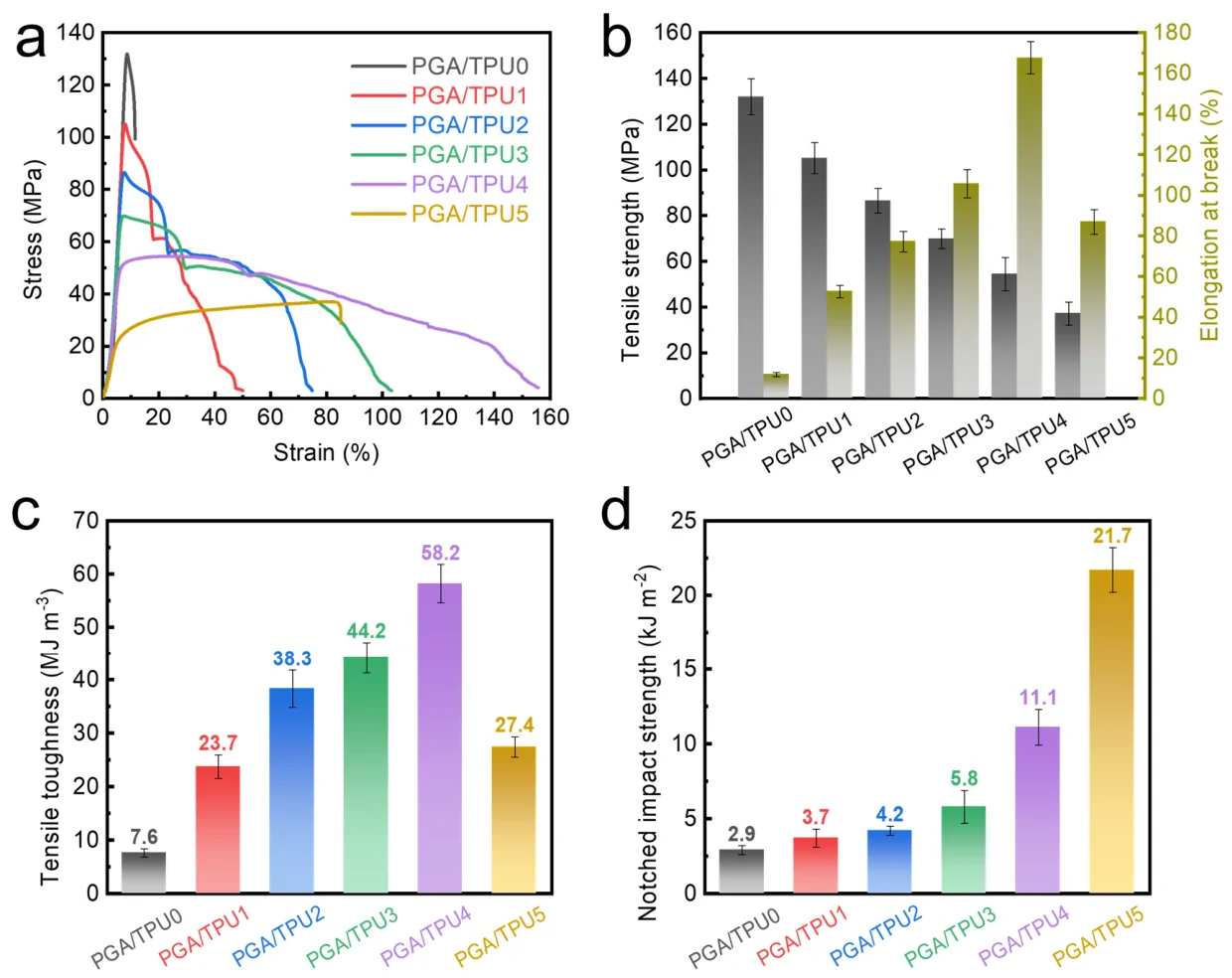
(**a**) Stress–strain curves of PGA/TPU blends; (**b**) Tensile strength and elongation at break; (**c**) Tensile toughness; (**d**) Notched impact strength.

Figure 4a displays the stress–strain curves of the PGA/TPU blends. Neat PGA exhibits a high tensile strength of 131.9 MPa and a low elongation at break of 12%. The absence of a yield plateau on the stress–strain curve indicates a typical brittle failure mode [24]. The tensile strength and elongation at break are summarized in Figure 4b and Table S5. The incorporation of the flexible TPU phase significantly improves the ductility of the matrix while inevitably sacrificing tensile strength. For instance, the tensile strength of the PGA/TPU4 blend decreases to 54.4 MPa, whereas its elongation at break increases markedly to 168%. This pronounced increase in elongation at break reflects the enhanced ductility of the blend and is accompanied by visible stress whitening during deformation, indicating a transition from brittle to ductile deformation.

Figure 4c and Table S5 present the tensile toughness of the blends, which represents the total energy absorbed during the tensile deformation process (area under the stress–strain curve). Neat PGA shows a low tensile toughness of 7.6 MJ m^−3^ because of its limited deformation before fracture. With increasing TPU content, the tensile toughness increases markedly and reaches a maximum of 58.2 MJ m^−3^ for the PGA/TPU4 blend. The increase in tensile toughness indicates that the blends can absorb more energy during tensile deformation before fracture. This improvement is associated with the enhanced ductility of the blends and the evolution of the phase morphology. With increasing TPU content, the dispersed TPU domains become larger and gradually evolve toward a co-continuous morphology, promoting more extensive plastic deformation of the PGA matrix. The appearance of a yield plateau at higher TPU contents further reflects the enhanced deformation capability of the blends.

Figure 4d and Table S5 display the notched Izod impact strength results. Neat PGA shows a low impact strength of 2.9 kJ m^−2^, whereas the incorporation of TPU substantially improves the impact resistance. In particular, the PGA/TPU5 blend reaches a notched impact strength of 21.7 kJ m^−2^, representing an approximate 650% increase compared to neat PGA. Under impact loading, the TPU phase promotes energy dissipation through mechanisms such as crazing, shear yielding, and interfacial debonding. At low TPU contents, crazing is more prominent, whereas at higher TPU contents, the enlarged elastomeric domains can impede crack propagation and promote more extensive plastic deformation of the surrounding PGA matrix.

Considering the subsequent reactive compatibilization study, PGA/TPU2 (80/20) was selected as a representative baseline composition rather than the composition with the maximum toughening efficiency. Although higher TPU contents (PGA/TPU4 and PGA/TPU5) provided higher elongation at break and impact strength, they were accompanied by a substantial decrease in tensile strength. In comparison, PGA/TPU2 exhibited a more balanced combination of tensile strength (86.5 MPa), elongation at break (77%), and tensile toughness (38.3 MJ m^−3^), making it suitable for evaluating the effect of ADR on interfacial regulation and strength–toughness balance.

### 3.2. Effect of ADR Content on Interfacial Modification of PGA/TPU Blends

#### 3.2.1. Processability of PGA/TPU/ADR Blends

**Figure 5:**
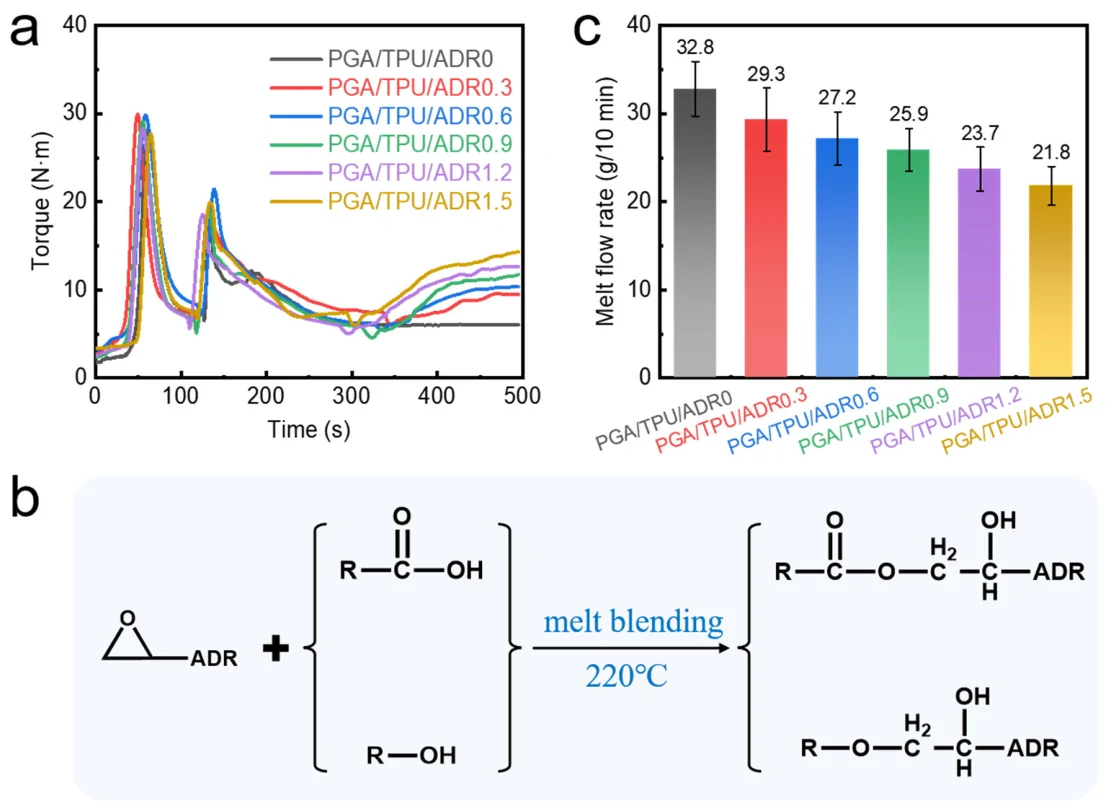
(**a**) Torque evolution versus time for PGA/TPU/ADR blends; (**b**) Proposed epoxy ring-opening reactions between ADR and terminal functional groups of PGA/TPU chains during melt processing; (**c**) MFR of the blends.

While the incorporation of the TPU phase effectively enhances the toughness of PGA, it inevitably leads to a reduction in tensile strength due to weak interfacial adhesion. Consequently, ADR was introduced during melt blending as a reactive compatibilizer to achieve a better balance between toughness and strength. Figure 5a displays the torque evolution of the PGA/TPU/ADR blends during compounding. The curves exhibit three characteristic torque responses associated with the initial melting of PGA pellets, the subsequent addition of the TPU phase, and the incorporation of ADR, respectively. The torque response becomes more pronounced with increasing ADR content. This behavior may be associated with the reactivity of the epoxy groups in ADR toward terminal carboxyl and hydroxyl groups of the PGA and TPU chains, which may promote chain extension, branching, and interfacial coupling reactions during melt processing (Figure 5b) [25,26]. These reactions may increase molecular architecture complexity and chain entanglement, thereby increasing the resistance to melt flow [27]. Consistent with the torque analysis, the MFR results presented in Figure 5c show a decreasing trend with increasing ADR content.

#### 3.2.2. Thermal Properties of PGA/TPU/ADR Blends

**Figure 6:**
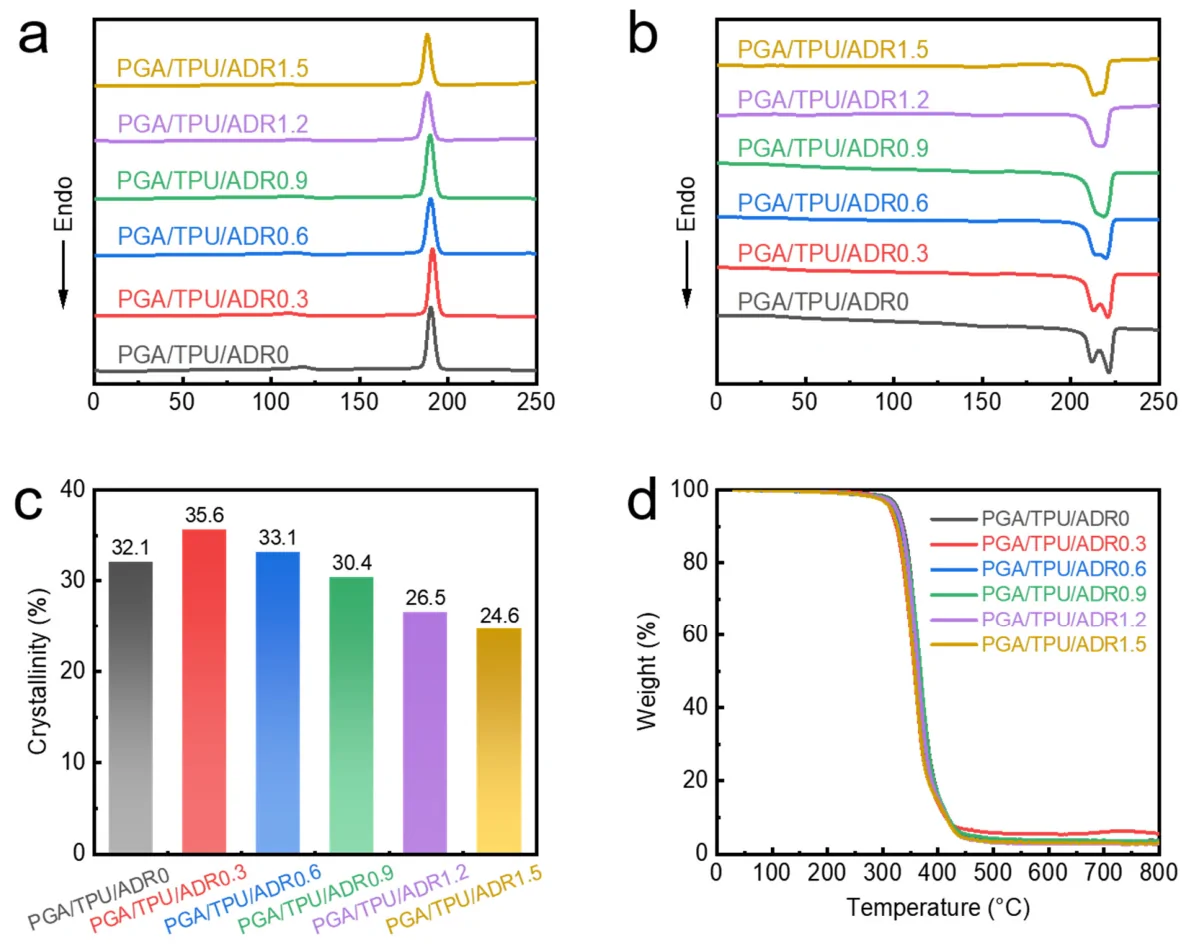
Thermal properties of PGA/TPU/ADR blends: (**a**) Cooling DSC curves; (**b**) Second heating DSC curves; (**c**) Crystallinity variations; (**d**) TGA thermograms.

The cooling DSC thermograms of the PGA/TPU/ADR blends are presented in Figure 6a and Table S6. It is observed that the *T_c_* of the PGA matrix shifts to lower values upon the addition of ADR. Specifically, the *T_c_* drops to 188.0 °C for the blend containing 1.5 phr ADR. This retardation in crystallization kinetics is attributed to ADR-induced chain extension and branching, which increase molecular architecture complexity, restrict segmental mobility, and hinder the rearrangement of PGA chains into a regular crystal lattice. Figure 6b displays the second heating DSC scans where a monotonic decrease in the *T_m_* of the PGA phase is evident. The *T_m_* decreases from 221.1 °C for the blend with 0.3 phr ADR to 213.5 °C for the blend with 1.5 phr ADR. This trend is consistent with ADR-induced chain extension and branching, which increase structural irregularity and restrict the regular packing of PGA chains. The resulting structural irregularity disrupts the perfection of the crystal lamellae and leads to a reduction in the melting point [28].

The variation in the *χ_c_* is depicted in Figure 6c and Table S6. Interestingly, the introduction of a small amount of ADR initially promotes crystallization. The *χ_c_* reaches a maximum of 35.6% for the blend containing 0.3 phr ADR. This enhancement is likely associated with the improved interfacial interactions and refined dispersed TPU morphology induced by ADR. These structural changes may facilitate PGA chain rearrangement and crystal growth by modifying the interfacial environment and phase morphology. However, as the ADR content further increases, the crystallinity begins to decline. For instance, *χ_c_* drops to 26.5% for the blend with 1.2 phr ADR. At higher ADR loadings, the increased degree of chain extension and branching may increasingly restrict the mobility of PGA chains and hinder their rearrangement into ordered crystalline structures, thereby suppressing the overall crystallization process. Figure 6d and Table S6 illustrate the thermal degradation profiles obtained from TGA. Although ADR enhances interfacial interactions between PGA and TPU, the accompanying increase in molecular architecture complexity may disturb the compact packing of the matrix chains. Consequently, the thermal stability of the ternary blends exhibits a slight decrease compared with the uncompatibilized binary blend.

#### 3.2.3. Morphology of PGA/TPU/ADR Blends

**Figure 7:**
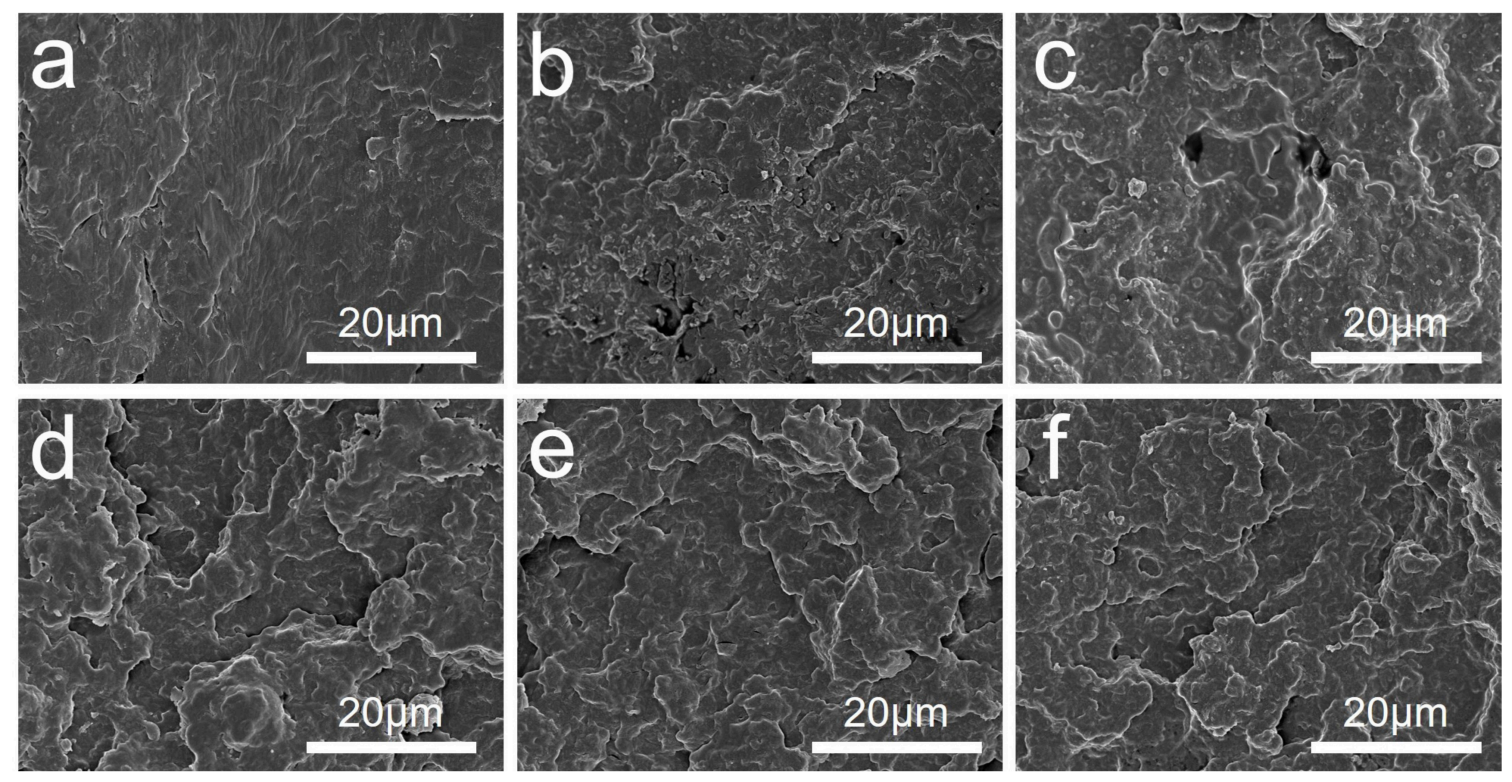
SEM micrographs of impact fracture surfaces for PGA/TPU/ADR blends with varying ADR contents: (**a**) PGA/TPU/ADR0; (**b**) PGA/TPU/ADR0.3; (**c**) PGA/TPU/ADR0.6; (**d**) PGA/TPU/ADR0.9; (**e**) PGA/TPU/ADR1.2; (**f**) PGA/TPU/ADR1.5.

Figure 7 presents the SEM micrographs of the impact fracture surfaces for the PGA/TPU/ADR blends. The introduction of ADR leads to a progressively refined dispersed-phase morphology and less distinct phase boundaries, suggesting enhanced interfacial interactions between the PGA matrix and the dispersed TPU phase. Consequently, a gradual and significant reduction in the domain size of the dispersed phase is observed with increasing ADR content. Compared with the binary blend without ADR, which displays relatively coarse domains, the ADR-containing systems exhibit a much finer phase dispersion. Such morphological refinement can influence the deformation and fracture behavior because the characteristic size of the dispersed phase is known to affect the relative contributions of crazing and shear yielding [29,30]. At higher ADR loadings such as 1.2 phr, the phase boundaries become increasingly indistinct within the matrix. This morphological evolution suggests that ADR-induced interfacial reactions suppress phase coalescence and enhance interphase interactions. Furthermore, the fracture surfaces of the ADR-containing blends exhibit greater roughness and more deformation-related cavities than the uncompatibilized blend. These features are consistent with more extensive plastic deformation and energy dissipation during fracture.

#### 3.2.4. Mechanical Properties of PGA/TPU/ADR Blends

**Figure 8:**
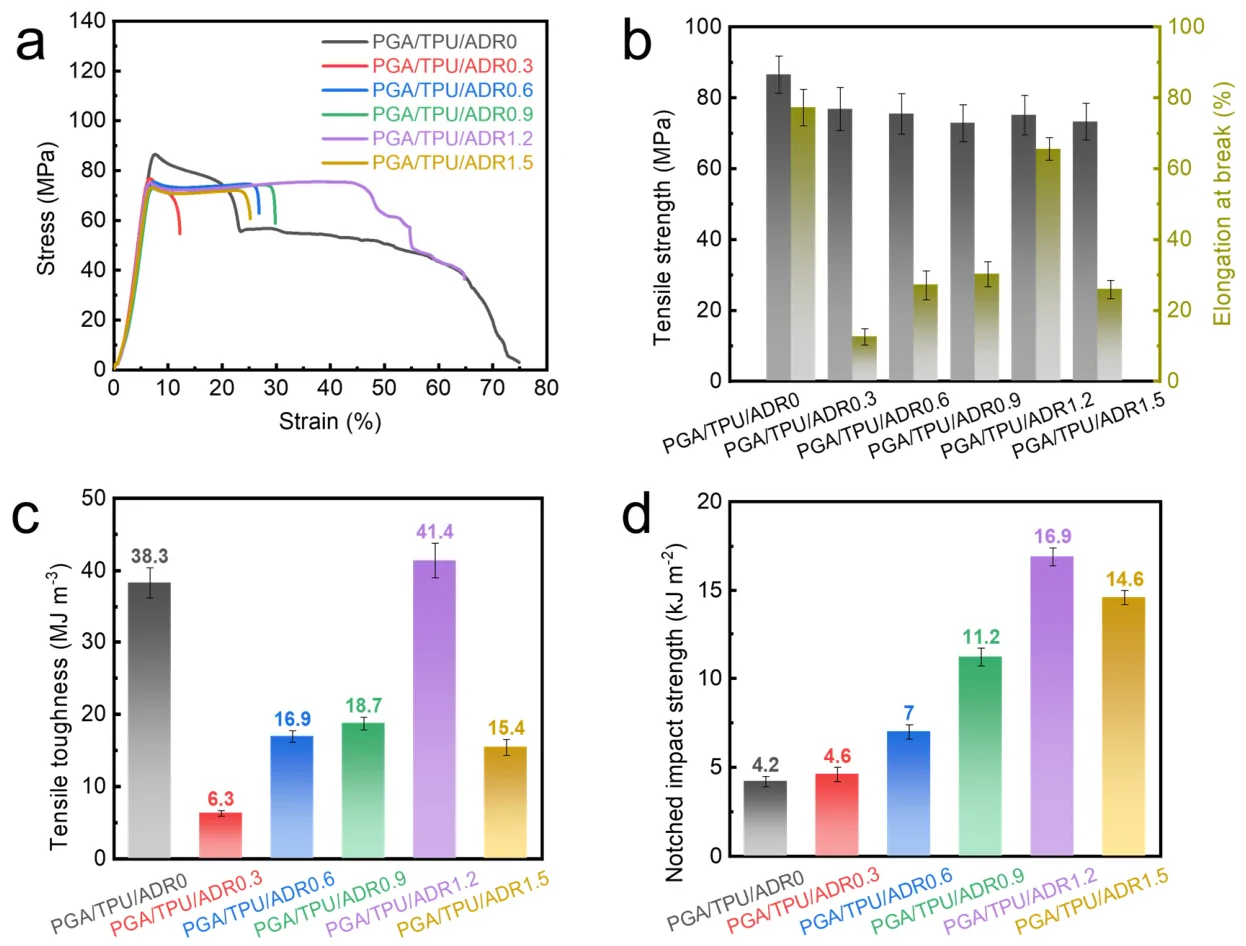
(**a**) Stress–strain curves of PGA/TPU/ADR blends; (**b**) Tensile strength and elongation at break; (**c**) Tensile toughness; (**d**) Notched impact strength.

Figure 8 presents the mechanical properties of the ternary PGA/TPU/ADR blends. As depicted in Figure 8a, the stress–strain behavior changes noticeably with the addition of ADR, and a more pronounced yield behavior is observed for some compatibilized compositions. This indicates enhanced plastic deformation capability compared with the uncompatibilized PGA/TPU blend. The tensile strength results are summarized in Figure 8b and Table S7. The addition of ADR causes a moderate decrease in tensile strength relative to the PGA/TPU/ADR0 blend. This behavior may be associated with ADR-induced changes in molecular architecture and crystallization behavior, together with possible plasticization by residual low-molar-mass species.

Figure 8c illustrates the tensile toughness of the blends, defined as the area under the tensile stress–strain curve [31]. The tensile toughness reaches 41.4 MJ m^−3^ for the blend containing 1.2 phr ADR. This value reflects the increased amount of energy absorbed during tensile deformation before fracture. The corresponding elongation at break reaches 65%, indicating improved ductility compared with the lower-ADR formulations. The enhanced tensile toughness is associated with the combined effects of improved interfacial interactions, refined phase morphology, and increased plastic deformation of the matrix.

Figure 8d displays the notched Izod impact strength results. At low ADR loadings such as 0.3 phr, the impact strength shows only a marginal increase to 4.6 kJ m^−2^. With increasing ADR content, the impact strength increases progressively and reaches 16.9 kJ m^−2^ at 1.2 phr ADR. This improvement indicates enhanced resistance to impact fracture. The improvement in impact resistance is therefore interpreted as arising from the combined effects of strengthened interfacial interactions and refined dispersed-phase morphology, which may facilitate stress transfer and promote more extensive plastic deformation and energy dissipation during impact. When the ADR content is further increased to 1.5 phr, the impact strength decreases, indicating that further ADR addition does not provide additional improvement in impact performance. This decline may be associated with the accompanying increase in melt-flow resistance and changes in phase morphology. Considering these mechanical properties collectively, PGA/TPU/ADR1.2 shows a favorable combination of tensile strength, tensile toughness, and notched impact strength within the investigated ADR range.

## 4. Conclusions

In this work, a poly(ε-caprolactone)-based TPU elastomer was synthesized and melt-blended with PGA, effectively enhancing the toughness of the rigid matrix. The investigation into the structure-property relationships revealed significant improvements in ductility and impact resistance for the binary systems; notably, the PGA/TPU4 blend achieved an elongation at break of 168% (approximately 14 times that of neat PGA), while the PGA/TPU5 blend reached a notched impact strength of 21.7 kJ m^−2^. To further optimize the interfacial adhesion and phase morphology, the epoxy-functionalized agent ADR was introduced as a reactive compatibilizer. Rheological and morphological analyses indicated that ADR promoted chain extension/branching and enhanced interfacial interactions, leading to refinement of the dispersed-phase morphology. Consequently, the compatibilized PGA/TPU/ADR1.2 blend achieved a robust notched impact strength of 16.9 kJ m^−2^ while maintaining excellent tensile toughness, demonstrating that the synergy of physical blending and reactive compatibilization offers a facile and effective strategy for fabricating high-performance PGA-based polymer blends with a balanced combination of strength and toughness.

## Data Availability

The data that support the findings of this study are available from the corresponding author upon reasonable request.

## Supplementary Materials

The following supporting information can be downloaded at: https://www.mdpi.com/article/10.3390/polym18202471/s1, Table S1. Formulation of the TPU. Table S2. Formulation of the PGA/TPU blends. Table S3 Formulation of the PGA/TPU/ADR blends. Table S4. Thermal performance data of the PGA/TPU blends. Table S5. Mechanical property data of the PGA/TPU blends. Table S6. DSC thermal performance data of the PGA/TPU/ADR blends. Table S7. Mechanical property data of the PGA/TPU/ADR blends. Figure S1. Schematic diagram of TPU synthetic route. Figure S2. FTIR spectrum of the TPU. Figure S3. Stress-strain curve of the TPU.
