# Supplementary material for: Reactive Compatibilization of Polyglycolic Acid/Polyurethane Blends for an Improved Strength–Toughness Balance
Source: Polymers (Basel). 2026 Oct 10;18(20):2471. doi: 10.3390/polym18202471 (PMC13709185; doi:10.3390/polym18202471)
Supplement: Supplementary file 1 [file polymers-18-02471-s001.zip › polymers-4560441-supplementary.pdf]

Supplementary Information

This file contains **Supporting Tables S1-S7 and Figs. S1-S3**:

Table S1 Formulation of the TPU

Table S2 Formulation of the PGA/TPU blends

Table S3 Formulation of the PGA/TPU/ADR blends

Table S4 Thermal performance data of the PGA/TPU blends

Table S5 Mechanical property data of the PGA/TPU blends

Table S6 DSC thermal performance data of the PGA/TPU/ADR blends

Table S7 Mechanical property data of the PGA/TPU/ADR blends

Fig. S1. Schematic diagram of TPU synthetic route.

Fig. S2. FTIR spectrum of the TPU.

Fig. S3. Stress-strain curve of the TPU.

Table S1 Formulation of the TPU

Name	Soft segment molecular weight (g mol^{-1})	Hard segment content (%)	Molar ratio of poly(ε-caprolactone):HDI:BDO
TPU	1250	45	1:4.31:3.31

Table S2 Formulation of the PGA/TPU blends

Name	PGA/TPU0	PGA/TPU1	PGA/TPU2	PGA/TPU3	PGA/TPU4	PGA/TPU5
PGA/wt%	100	90	80	70	60	50
TPU/ wt%	0	10	20	30	40	50

Table S3 Formulation of the PGA/TPU/ADR blends

Name	PGA/TPU/ ADR 0	PGA/TPU/ ADR0.3	PGA/TPU/ ADR0.6	PGA/TPU/ ADR0.9	PGA/TPU/ ADR1.2	PGA/TPU/ ADR1.5
ADR/phr	0	0.3	0.6	0.9	1.2	1.5

Matrix composition (wt%): PGA/TPU = 80/20

Table S4 Thermal performance data of the PGA/TPU blends

Name	T_c (°C)	T_m (°C)	ΔH_m (J/g)	χ_c (%)	$T_{5\%}$ (°C)
PGA/TPU0	187.9	220.8	59.5	30.0	341.7
PGA/TPU1	190.3	221.8	54.0	30.3	346.9
PGA/TPU2	190.9	221.7	50.9	32.1	341.2
PGA/TPU3	191.4	221.5	45.8	33.0	335.8
PGA/TPU4	191.5	222.0	41.1	34.5	334.4
PGA/TPU5	193.1	220.6	35.4	35.7	321.9

Table S5 Mechanical property data of the PGA/TPU blends

Name	Tensile strength (MPa)	Elongation at break (%)	Tensile toughness (MJ m^{-3})	Notched impact strength (kJ m^{-2})
PGA/TPU0	131.9 ± 7.9	12 ± 1	7.6 ± 0.8	2.9 ± 0.3
PGA/TPU1	105.1 ± 6.8	53 ± 3	23.7 ± 2.2	3.7 ± 0.6
PGA/TPU2	86.5 ± 5.4	77 ± 5	38.3 ± 3.5	4.2 ± 0.3
PGA/TPU3	69.8 ± 4.3	106 ± 7	44.2 ± 2.9	5.8 ± 1.1
PGA/TPU4	54.4 ± 7.3	168 ± 8	58.2 ± 3.6	11.1 ± 1.2
PGA/TPU5	37.1 ± 5	87 ± 6	27.4 ± 1.9	21.7 ± 1.5

Table S6 DSC thermal performance data of the PGA/TPU/ADR blends

Name	T_c (°C)	T_m (°C)	ΔH_m (J g^{-1})	χ_c (%)	$T_{5\%}$ (°C)
PGA/TPU/ADR0	190.9	221.7	50.9	32.1	341.2
PGA/TPU/ADR0.3	191.1	221.1	56.4	35.6	326.1
PGA/TPU/ADR0.6	189.8	219.7	52.5	33.1	336.4
PGA/TPU/ADR0.9	189.6	219.0	48.2	30.4	341.1
PGA/TPU/ADR1.2	188.2	216.3	42.0	26.5	338.1
PGA/TPU/ADR1.5	188.0	213.5	38.9	24.6	332.1

Table S7 Mechanical property data of the PGA/TPU/ADR blends

Name	Tensile strength (MPa)	Elongation at break (%)	Tensile toughness (MJ m^{-3})	Notched impact strength (kJ m^{-2})
PGA/TPU/ADR0	86.5 ± 5.3	77 ± 5	38.3 ± 2.1	4.2 ± 0.3
PGA/TPU/ADR0.3	76.8 ± 6.1	12 ± 2	6.3 ± 0.4	4.6 ± 0.4
PGA/TPU/ADR0.6	75.4 ± 5.7	27 ± 4	16.9 ± 0.8	7.0 ± 0.4
PGA/TPU/ADR0.9	72.8 ± 5.2	30 ± 3	18.7 ± 0.9	11.2 ± 0.5
PGA/TPU/ADR1.2	75.1 ± 5.6	65 ± 3	41.4 ± 2.4	16.9 ± 0.5
PGA/TPU/ADR1.5	73.2 ± 5.2	26 ± 2	15.4 ± 1.1	14.6 ± 0.4

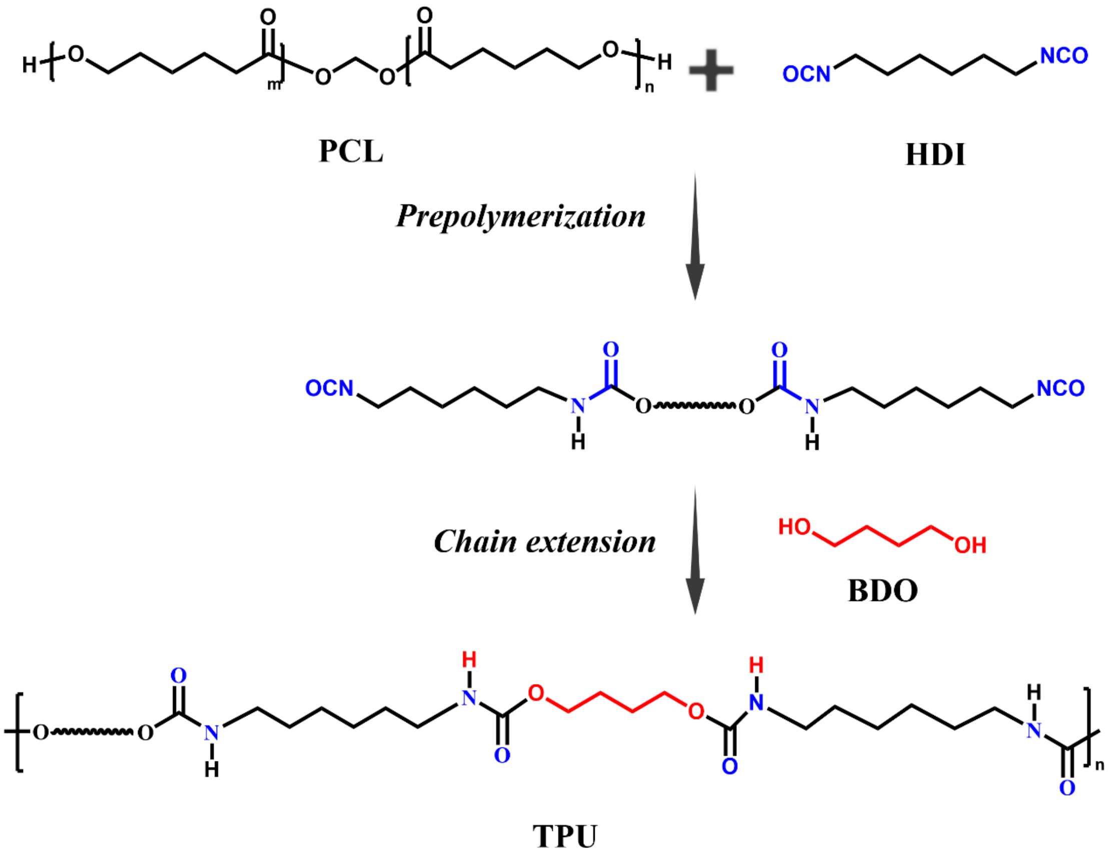

Fig. S1. Schematic diagram of the TPU synthetic route.

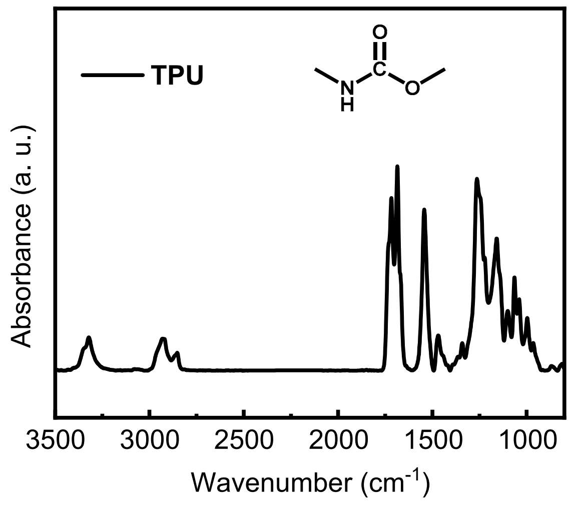

Fig. S2. FTIR spectrum of the TPU.

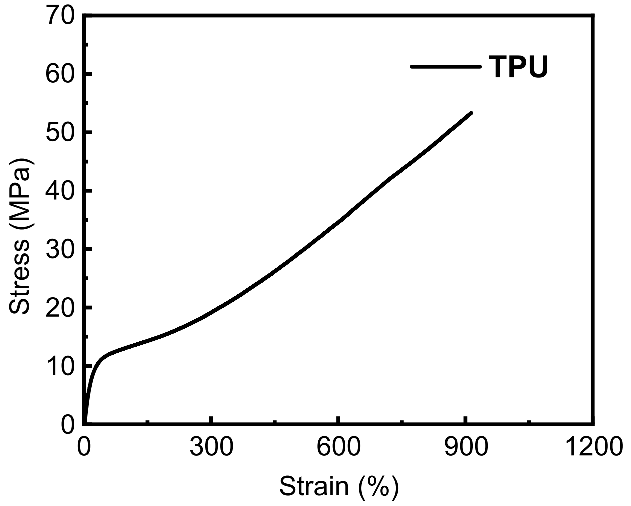

Fig. S3. Stress-strain curve of the TPU.
